# CRISPR/Cas9 Delivery Mediated with Hydroxyl‐Rich Nanosystems for Gene Editing in Aorta

**DOI:** 10.1002/advs.201900386

**Published:** 2019-04-20

**Authors:** Xiaoping Zhang, Chen Xu, Shijuan Gao, Ping Li, Yu Kong, Tiantian Li, Yulin Li, Fu‐Jian Xu, Jie Du

**Affiliations:** ^1^ State Key Laboratory of Chemical Resource Engineering Key Lab of Biomedical Materials of Natural Macromolecules (Beijing University of Chemical Technology) Ministry of Education Beijing Laboratory of Biomedical Materials, and Beijing Advanced Innovation Center for Soft Matter Science and Engineering Beijing University of Chemical Technology Beijing 100029 China; ^2^ Key Laboratory of Remodeling‐Related Cardiovascular Diseases (Ministry of Education), and Beijing Institute of Heart, Lung and Blood Vessel Diseases Beijing Anzhen Hospital Affiliated to Capital Medical University Beijing 100029 China

**Keywords:** aorta disease, cationic carriers, CRISPR‐associated nuclease 9 delivery, genome editing, hydroxyl‐rich

## Abstract

A CRISPR/Cas9 system has emerged as a powerful tool for gene editing to treat genetic mutation related diseases. Due to the complete endothelial barrier, effective delivery of the CRISPR/Cas9 system to vasculatures remains a challenge for in vivo gene editing of genetic vascular diseases especially in aorta. Herein, it is reported that CHO‐PGEA (cholesterol (CHO)‐terminated ethanolamine‐aminated poly(glycidyl methacrylate)) with rich hydroxyl groups can deliver a plasmid based pCas9‐sg*Fbn*1 system for the knockout of exon 10 in *Fbn*1 gene. This is the first report of a polycation‐mediated CRISPR/Cas9 system for gene editing in aorta of adult mice. CHO‐PGEA/pCas9‐sg*Fbn*1 nanosystems can effectively contribute to the knockout of exon 10 in *Fbn*1 in vascular smooth muscle cells in vitro, which leads to the change of the phosphorylation of Smad2/3 and the increased expression of two downstream signals of *Fbn*1: *Mmp‐*2 and *Ctgf*. For in vivo application, the aortic enrichment of CHO‐PGEA/Cas9‐sg*Fbn*1 is achieved by administering a pressor dose of angiotensin II (Ang II). The effects of the pCas9‐sg*Fbn*1 system targeting *Fbn*1 demonstrate an increase in the expression of *Mmp‐*2 and *Ctgf* in aorta. Thus, the combination of CHO‐PGEA/pCas9‐sg*Fbn*1 nanosystems with Ang II infusion can provide the possibility for in vivo gene editing in aorta.

## 1 Introduction

Gene editing technology based on the clustered regularly interspaced short palindromic repeat/CRISPR‐associated nuclease 9 (CRISPR/Cas9) has great potential for treatment of genetic diseases.^1^, ^2^, ^3^, ^4^ The CRISPR/Cas9 system consists of two critical components: the single‐guide RNA (sgRNA) and Cas9 nuclease.^5^, ^6^ The sgRNA is complexed with Cas9 protein to target the genome to introduce breaks in a specific DNA sequence.^7^, ^8^ The breaks are repaired by introducing a sequence to induce the destruction of the sequence or promote the replacement for a healthy target in the presence of donor template DNA.^2^, ^9^ Due to its simplicity, high specificity, and efficiency, the CRISPR/Cas9 system has already been widely used to edit genes in various cell lines.^10^, ^11^

Although CRISPR/Cas9 system has widely used in vitro, the effective delivery of CRISPR/Cas9 system remains a challenge for in vivo applications. Both viral and nonviral approaches are being evaluated for in vivo CRISPR/Cas9 delivery.^1^, ^12^, ^13^, ^14^, ^15^ Viral delivery, such as adeno‐associated viral vector, is promising for gene delivery but may suffer from limited packaging capacity.^16^ In comparison, nonviral nanoparticles have the potential to overcome the limitations of large packaging capacity and serve as efficient and alternative DNA carriers due to their special structures, biocompatibility, and good membrane permeability.^17^, ^18^ Some nonviral vectors, for example, liposomes,^19^ polycations,^20^ and inorganic nanoparticles,^9^ were proved to be capable of delivering CRISPR/Cas9 system for gene editing. In particular, nonviral vector‐mediated CRISPR/Cas9 system has made a series of advances in tumors due to the enhanced permeability and retention effect.^18^, ^21^ Expression of genetic mutations in vascular smooth muscle cells (VSMCs) plays an important role in vascular diseases, thus VSMCs can be served as the key target cells for gene editing. However, VSMCs exhibit very low transfection efficiency.^22^, ^23^ Thus, the efficient delivery of CRISPR/Cas9 system to VSMCs and aorta tissue is a major obstacle to overcome.

Hydroxyl‐rich gene vectors were appealing due to their low cytotoxicity and high transfection efficiency.^24^, ^25^, ^26^ Such hydroxyl‐rich gene vector has great potential for the treatment of heart disease and tumors.^27^, ^28^, ^29^ We recently reported that the cholesterol (CHO)‐terminated ethanolamine‐aminated poly(glycidyl methacrylate) (CHO‐PGEA) with rich hydroxyl groups has great stability and high transfection efficiency for gene delivery in tumor.^30^ Moreover, lipid‐based carriers can effectively enhance the transfection efficiency because lipids are the important components of cell membranes.^31^, ^32^ We thus hypothesized that the CHO‐PGEA with amino and hydroxyl‐rich units could complex the “all‐in‐one” plasmid carrying both the Cas9 and sgRNA expression cassettes to perform genome editing in aorta. To demonstrate a proof of concept of CHO‐PGEA mediated CRISPR/Cas9 delivery and in vivo gene editing, we chose the Marfan syndrome as a gene editing disease model. Marfan syndrome is a common autosomal dominant disorder of connective tissue and an inherited lethal disease,^33^, ^34^ which is mainly caused by mutations in *Fbn*1 that encodes fibrillin‐1.^35^, ^36^

**Figure 1:**
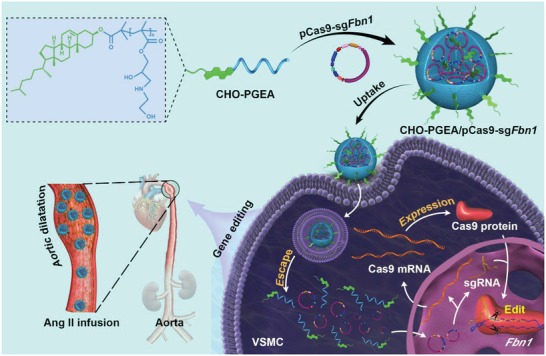
Schematic illustration of the formation of CHO‐PGEA/pCas9 nanoparticles and the angiotensin II (Ang II)‐assisted in vivo delivery for efficient gene editing in adult mouse aorta.

Normal vascular tissue has a complete endothelial barrier to resist invasion of exogenous substances.^37^ Angiotensin II (Ang II) could increase vascular wall pressure and enhance vascular permeability.^38^, ^39^ We have previously shown that a dose of Ang II could promote polycation/miRNA complex to pass through vascular endothelium and increase transfection efficiency.^27^ In this work, we build an Ang II‐assisted CHO‐PGEA/Cas9 delivery nanosystem for *Fbn*1 editing in adult mouse aorta (**Figure** **1**). Polycation‐mediated CRISPR/Cas9 system was first time demonstrated to achieve the efficient gene editing in VSMCs and aorta, which would provide a novel tool for the study of casual mutation in vivo and the therapy for genetic vascular diseases.

## 2 Results and Discussions

### 2.1 Construction and Validation of CRISPR/Cas9 Plasmid Targeting *Fibrillin‐*1

**Figure 2:**
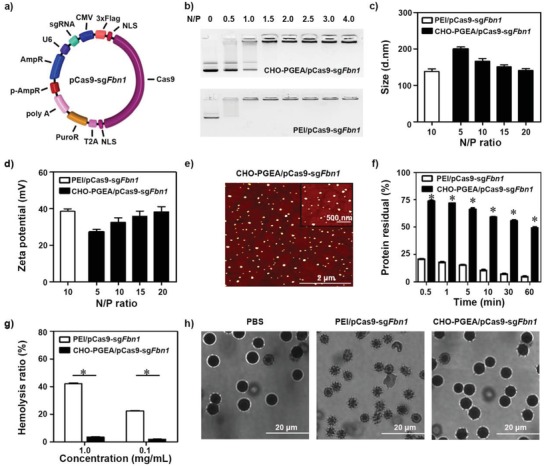
a) Schematic diagram of pCas9‐sg*Fbn*1 plasmid. b) Agarose gel electrophoresis. c) Particle sizes and d) zeta potentials of polycation/pCas9‐sg*Fbn*1 complexes at various N/P ratios. e) Morphologies of CHO‐PGEA/pCas9‐sg*Fbn*1 nanoparticles at N/P = 15 measured by atomic force microscope. f) Protein assay of polycation/pCas9‐sg*Fbn*1 complexes (at their respective N/P ratio) treated with excess BSA. g) Hemolysis ratio of red blood cells (RBCs) treated with different polycaiton/pCas9‐sg*Fbn*1 complexes at the concentration of 0.1 and 1 mg mL^−1^, where deionized water and PBS were used as positive and negative controls. h) Morphologies of RBCs treated with PBS, PEI/pCas9‐sg*Fbn*1, and CHO‐PGEA/pCas9‐sg*Fbn*1 (*P < 0.05; data are from three independent experiments).

To construct the Cas9‐sg*Fbn*1 system, we chose sgRNA targeting the exon 10 of *Fbn*1 gene because in this exon we identified a premature termination codon mutation from aortic aneurysm patients. Three sgRNAs targeting the exon 10 of *Fbn*1 were designed by using the MIT website (http://crispr.mit.edu/) (Figure S1a in the Supporting Information). Then we individually cloned these three sgRNAs (sgRNA1, sgRNA2, sgRNA3) into pX459 vector, which coexpresses one sgRNA and Cas9 nuclease (**Figure** **2**a). These three sgRNAs exhibited targeting efficiency ≈35% based on the T7 endonuclease I (T7EI) assay (Figure S1b in the Supporting Information). Next, the MIT website was used to search for potential off‐target sequences of these three sgRNAs. Among the three sgRNAs, the sgRNA3 showed less off‐target effects with only four potential off‐target sites on open reading frame whose sequences are similar to the target sequence but contain 3‐4‐base mismatches (Figure S2a in the Supporting Information). Therefore, the plasmid which coexpresses sgRNA3 and Cas9 nuclease (named pCas9‐sg*Fbn*1) was selected for further characterization.

Then, it was examined whether pCas9‐sg*Fbn*1 can cleave target site in vitro. The DNA sequencing for polymerase chain reaction (PCR) amplification that exceeds the boundaries of the targeted sites showed two or more peaks at the same position after pCas9‐sg*Fbn*1 treatment, indicating that pCas9‐sg*Fbn*1 could induce gene editing near the target site (Figure S1c in the Supporting Information). Furthermore, the short deletions at 3–5 bases upstream of the protospacer adjacent motif were observed, confirming the specificity of this targeting process (Figure S1d in the Supporting Information). The mutation frequency was ≈28 %. Most mutations were the deleted ones (1 bp, 2 bp, etc.), which resulted in the loss of gene function (Figure S1d in the Supporting Information). Meanwhile, the potential off‐target sites were amplified from the cells transfected with pCas9‐sg*Fbn*1 followed by DNA sequencing. No off‐target mutations which were predicted using the MIT website were detected (Figure S2b in the Supporting Information). Collectively, pCas9‐sg*Fbn*1 plasmid constructed here can target *Fbn*1 and induce deleted mutations in cells with high efficiency and specificity.

### 2.2 Preparation and Characterization of CHO‐PGEA/pCas9‐sg*Fbn*1 Nanoparticles

Due to the large size of the CRISPR/Cas9 system (≈9 kb), its efficient in vivo delivery is the critical element to achieve effective gene editing. Our recently published work indicated that the introduction of cell membrane lipid molecules such as cholesterol (CHO) into polycations benifited transfection performances in vitro and in vivo.^30^ In this work, CHO‐PGEA was prepared based on CHO‐terminated poly(glycidyl methacrylate) (CHO‐PGMA, *M*_n_ = 9.25 × 10^3^ g mol^−1^) as we reported before.^30^ Then, we evaluated the potential of CHO‐PGEA for the delivery of plasmid‐based CRISPR/Cas9 system.

The physicochemical properties of the CHO‐PGEA/pCas9‐*sgFbn*1 nanoparticles including complexation ability, particle size, and zeta potential were tested and optimized for in vivo delivery. The agarose gel retardation assay showed that CHO‐PGEA bound to the Cas9‐sg*Fbn*1 plasmid efficiently and delayed plasmid migration at the N/P ratio of 1.5 (Figure 2b). The sizes and zeta potentials of the CHO‐PGEA/pCas9‐sg*Fbn*1 nanoparticles were detected by dynamic light scattering, where branched polyethylenimine (PEI) (25 kDa) was as a golden standard at its optimal N/P ratio of 10.^30^ The particle sizes of all the nanoparticles ranged from 150 to 200 nm at various N/P ratios (Figure 2c) with uniform distribution (Table S1 in the Supporting Information). There is no significant change in particle size when N/P ratio was more than 15. CHO‐PGEA actually achieved higher transfection performance at the optimal N/P ratio of 15 (see below). The zeta potentials of the polycation/pCas9‐sg*Fbn*1 nano‐particles ranged from 25 to 40 mV (Figure 2d). Positive potentials of nanoparticles can enhance cellular uptake. Different particles were also visualized by atomic force microscopy (AFM) (Figure 2e; Figure S3 in the Supporting Information). All the generated polycation/pCas9‐sg*Fbn*1 nanoparticles showed the compact spherical morphologies.

When positively charged nanoparticles were administered in the body, they would be rapidly interacted by negatively charged molecules such as proteins in blood,^40^, ^41^ which may lead to aggregation.^42^ Therefore, less protein adsorption on nanoparticles and biocompatibility are very important for in vivo delivery. Bovine serum albumin (BSA), one of abundant proteins in plasma,^43^ was used to examine the ability of protein attachment onto the nanoparticles. PEI/pCas9‐sg*Fbn*1 nanoparticles at the optimal N/P ratio of 10 exhibited high protein adsorption, resulting in less than 20% free BSA after 1 min of treatment (Figure 2f). By contrast, CHO‐PGEA/pCas9‐sg*Fbn*1 nanoparticles at the optimal N/P ratio of 15 showed that significantly lower protein adsorption and 50% free BSA remain even after 1 h incubation. The unique resistance characteristics of protein adsorption are beneficial for in vivo gene delivery.

Hemolytic property was used to assess the biocompatibility of nanoparticles.^44^ PEI/pCas9‐sg*Fbn*1 nanoparticles exhibited one significantly higher hemolysis ratio (≈40%) at a concentration of 1 mg mL^−1^, while the hemolysis ratio of CHO‐PGEA/pCas9‐sg*Fbn*1 nanoparticles was just ≈3% (Figure 2g). Meanwhile, the amount of released hemoglobin also revealed that CHO‐PGEA/pCas9‐sg*Fbn*1 nanoparticles had much weaker hemolysis properties than PEI/pCas9‐sg*Fbn*1 nanoparticles (Figure S4 in the Supporting Information). The red blood cell treated with CHO‐PGEA/pCas9‐sg*Fbn*1 nanoparticle did not cause significant morphological change (Figure 2h). These excellent abilities of CHO‐PGEA were attributed to a large number of hydrophilic hydroxyl groups, which could shield the surface against the deleterious effects of excess positive charges.^26^, ^27^, ^28^

### 2.3 Cas9 Plasmid Transfection In Vitro and Cellular Uptake

**Figure 3:**
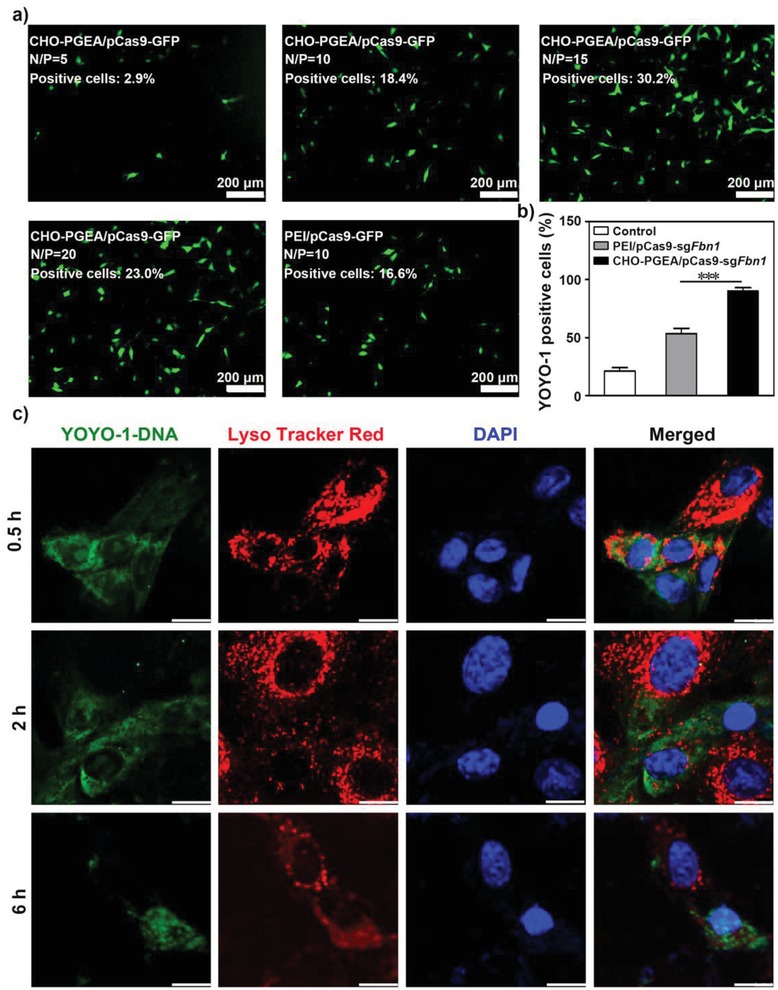
a) Fluorescence images of GFP expression mediated by CHO‐PGEA at different N/P ratios and PEI (at the optimal N/P ratio of 10) in mouse vascular smooth muscle cells (VSMCs). b) Flow cytometry analysis for cellular uptake efficiency of VSMCs incubated with CHO‐PGEA/pCas9‐sg*Fbn*1 and PEI/pCas9‐sg*Fbn*1 at their optimal N/P ratios. c) Fluorescence images of VSMCs treated with CHO‐PGEA/pCas9‐sg*Fbn*1 at 0.5, 2, and 6 h respectively. Green: YOYO‐1‐labeled pCas9‐sg*Fbn*1; Red: Lyso Tracker Red‐labeled the endosomes and lysosomes; Blue: DAPI‐labeled nuclei. The scale bar indicates 10 µm. ***P < 0.001.

To test whether CRISPR/Cas9 plasmid delivered by CHO‐PGEA at different N/P ratios could express in VSMCs, the plasmid carrying the green fluorescent protein (GFP)‐fused pCas9 was used to visualize Cas9 expression in VSMCs (**Figure** **3**a). The transfection efficiencies of CHO‐PGEA at the N/P ratios of 15 and 20 by flow cytometry were ≈30% and 23%, which is higher than that of PEI at its optimal N/P ratio of 10 (Figure 3a; Figure S5 in the Supporting Information). Figure S6 in the Supporting Information showed that the cytotoxicity was increased with the increase of N/P ratio. However, CHO‐PGEA exhibited much lower cytotoxicity than PEI at different N/P ratios, which was consistent with our earlier work.^30^ Considering both Cas9 plasmid transfection and cytotoxicity, the optimal ratio N/P of 15 was used to detect the potential of CHO‐PGEA to deliver CRISPR/Cas9 system at the following experiments.

Efficient cellular uptake is another important property for gene transfection.^31^ The cellular uptake efficiency of nanoparticles was assessed by delivering YOYO‐1‐labeled Cas9‐sg*Fbn*1 plasmid in VSMCs. Flow cytometry analysis showed that CHO‐PGEA/pCas9‐sg*Fbn*1 nanoparticles induced much higher YOYO‐1 positive cells (≈90%) in comparison with PEI/pCas9‐sg*Fbn*1 (Figure 3b; Figure S7 in the Supporting Information). The higher ability of CHO‐PGEA/pCas9‐sg*Fbn*1 nanoparticles to overcome cellular membranes barriers could be explained by the introduction of CHO, which might readily change membrane fluidity and increase biocompatibility.^30^

Effective endosome escape is a key characteristic for nanoparticle‐mediated gene expression. The intracellular distribution of CHO‐PGEA/pCas9‐sg*Fbn*1 nanoparticles was investigated. As shown in Figure 3c, the nanoparticles could bind to the cell surface after 0.5 h post transfection. The nanoparticles entered the cytosol, localized in the whole cytoplasm, and showed the colocalization with Lyso Tracker‐stained endosomes after 2 h post transfection. A amount of Cas9‐sg*Fbn*1 plasmids effectively escaped from the lysosome and penetrated into the nucleus after 6 h of incubation (Figure 3c). The above results indicated the effective endosome escape and successful release of Cas9‐sg*Fbn*1 plasmids from the nanoparticles.

### 2.4 CHO‐PGEA Delivery of pCas9‐sg*Fbn*1 for In Vitro Gene Disruption

**Figure 4:**
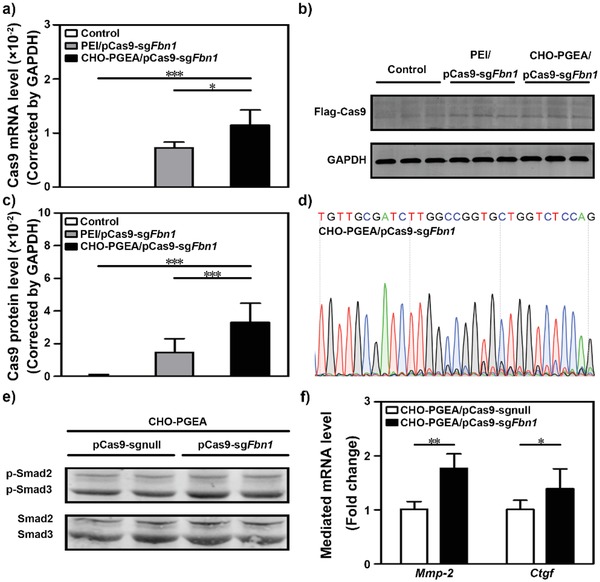
a) Real‐time PCR analysis for Cas9 mRNA expression in VSMCs treated as indicated. b,c) Western blot analysis of Cas9 protein expression in VSMCs incubated with nanoparticles with an anti‐Flag antibody. d) Sanger sequencing of PCR amplicon of the targeted *Fbn*1 locus in CHO‐PGEA/pCas9‐sg*Fbn*1 treated VSMCs. e) Western blot analysis of *Fbn*1 induced phosphorylation of Smad2/3 (p‐Smad2/3) in VSMCs after treatment with CHO‐PGEA/pCas9‐sg*Fbn*1 or CHO‐PGEA/pCas9‐sgnull. f) The mRNA levels of *Fbn*1 targeted genes *Mmp‐*2 and *Ctgf* in VSMCs after the treatments with CHO‐PGEA/pCas9‐sg*Fbn*1 and CHO‐PGEA/pCas9‐sgnull. *P < 0.05, **P < 0.01, and ***P < 0.001.

Inspired by these results, the potency of gene editing mediated by CHO‐PGEA/pCas9‐sg*Fbn*1 was tested. The Cas9 expression of pCas9‐sg*Fbn*1 was successfully detected by real‐time PCR (RT‐PCR) for Cas9 mRNA and western blot assays for Cas9 protein (**Figure** **4**a–c; Figure S8 in the Supporting Information). Consistent with the above results of in vitro pCas9 transfection (Figure 3a), CHO‐PGEA/pCas9‐sg*Fbn*1 demonstrated much higher level of Cas9 expression in comparison with PEI/pCas9‐sg*Fbn*1.

Upon the treatment of CHO‐PGEA/pCas9‐sg*Fbn*1, the DNA sequencing of the target sites revealed two or more peaks near the targeted site at the same position in comparison with CHO‐PGEA/pCas9‐sgnull (Figure 4d; Figure S9 in the Supporting Information). Thus, CHO‐PGEA/pCas9‐sg*Fbn*1 could induce gene editing near the target *Fbn*1 site. Then, it was investigated whether the gene disruption would lead to the loss of function of *Fbn*1. *Fbn*1, a large extracellular matrix glycoprotein,^35^, ^45^ functions as a negative regulator of transforming growth factor (TGF)‐β by forming a complex.^46^ Smad2/3 are the essential signaling molecules of canonical TGF‐β signaling pathway. The loss of function of *Fbn*1 leads to the release of active TGF‐β, which stimulates Smad phosphorylation and promotes the target genes expression of Smad.^47^, ^48^ *Fbn*1 mutations often lead to the abnormal activation of TGF‐β signaling pathways. Therefore, the activation of Smad2/3 was first evaluated. As expected, CHO‐PGEA/pCas9‐sg*Fbn*1 increased the phosphorylation of Smad2 and Smad3 (Figure 4e; Figures S10 and S11 in the Supporting Information). Next, the expression of Smad target genes including *Mmp‐*2 and *Ctgf* was examined. Similar to the increased Smad2/3 activation, the expression levels of *Ctgf* and *Mmp‐*2 were significantly elevated in the CHO‐PGEA/pCas9‐sg*Fbn*1 group (Figure 4f). These above results indicated that CHO‐PGEA could mediate Cas9‐sg*Fbn*1 plasmid to achieve *Fbn*1 gene disruption.

### 2.5 CHO‐PGEA Delivery of pCas9‐sg*Fbn*1 for In Vivo Gene Disruption

**Figure 5:**
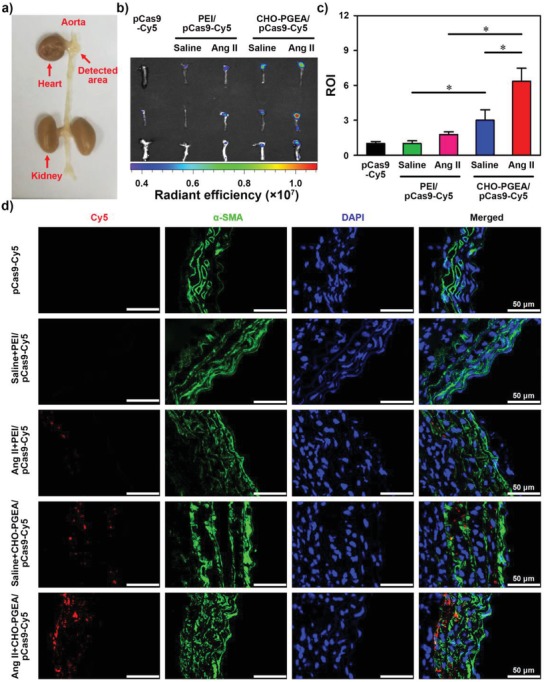
a) Cardiovascular image with the detecting area. b) Representative images and c) relative ROI of Ang II‐mediated or saline‐mediated mouse aorta after the accumulation of pCas9‐Cy5 and polycation/pCas9‐Cy5 nanoparticles determined by Xenogen IVIS imaging system. d) Fluorescence images of Ang II‐mediated or saline‐mediated mouse aortic sections at various treatments (*P < 0.05).

Because of blood flushing, the enrichment amounts of nanoparticles on aortic tissues are relatively low. Our previous work showed that a pressor dose of Ang II can enhance vascular permeability and promote nanoparticles to transmigrate endothelium.^27^ It was hypothesized that Ang II infusion can increase the enrichment of nanoparticles in aorta. To investigate the aortic delivery situation of CHO‐PGEA/pCas9‐sg*Fbn*1 nanoparticles in vivo, we preinfused mice with Ang II at 1500 ng kg^−1^ per min for 7 days. Cas9‐sg*Fbn*1 plasmids were labeled using the Cy5 molecular probe according to the instruction (named pCas9‐Cy5). The mice were treated with pCas9‐Cy5, PEI/pCas9‐Cy5, or CHO‐PGEA/pCas9‐Cy5. After 1 h, the enrichment of pCas9‐Cy5 in aorta was imaged (**Figure** **5**a). As shown in Figure 5b, free pCas9‐Cy5 hardly entered into the mouse aorta, because free pCas9‐Cy5 is easily degraded by nucleases in blood. Although PEI could effectively condense pCas9 (Figure 2b), the PEI/pCas9‐Cy5 fluorescence signal in aorta was evidently not detected, probably due to its poor antiprotein adsorption capacity in blood (Figure 2f). In comparison with PEI/pCas9‐Cy5 and free pCas9‐Cy5, CHO‐PGEA/pCas9‐Cy5 demonstrated much stronger fluorescent signals in the aorta (Figure 5b). Interestingly, there existed considerable differences in enrichments of CHO‐PGEA/pCas9‐Cy5 nanoparticles between the saline‐infused and Ang II‐infused groups (Figure 5c).

In addition, the aortic tissue sections of different groups were utilized to observe the cellular uptake of pCas9‐Cy5 by confocal microscope, where VSMCs were stained in green by immunofluorescence staining of α‐smooth muscle actin (SMA) and nuclei were stained by 4ʹ,6‐diamidino‐2‐phenylindole (DAPI) in blue (Figure 5d). No obvious fluorescence signal was observed in the free pCas9‐Cy5 and the PEI/pCas9‐Cy5 groups. Significant fluorescence signals were observed in the CHO‐PGEA/pCas9‐Cy5 group. In comparison with the saline infusion group, Ang II infusion further enhanced the enrichment of CHO‐PGEA/pCas9‐Cy5 in the aorta, which has the same trend as the result of Figure 5b. The above results indicated that CHO‐PGEA can effectively mediate the delivery of nucleic acid molecules into aorta. Very importantly, the delivery of CHO‐PGEA/pCas9‐Cy5 nanoparticles was more effective in Ang II‐infused aorta.

**Figure 6:**
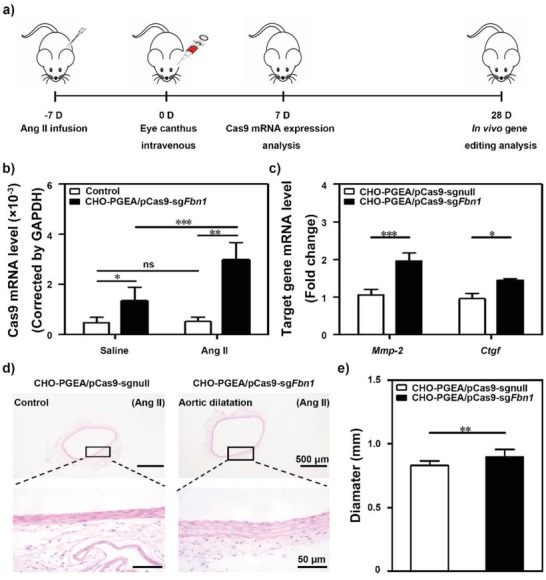
a) Key time points of different treatments in mouse. b) Real‐time PCR analysis for in vivo Cas9 levels in adult mice aortic tissues with or without Ang II treatment followed by intravenous injection of CHO‐PGEA/pCas9‐sg*Fbn*1. c) Relative expression of *Fbn*1 targeted genes *Mmp‐*2 and *Ctgf* mRNAs in mice aortic tissues. d,e) The aortic diameters were measured by H&E staining in the CHO‐PGEA/pCas9‐sgnull and CHO‐PGEA/pCas9‐sg*Fbn*1 groups. *P < 0.05, **P < 0.01, ***P < 0.001, and ns, no significant difference.

In order to assess the expression of pCas9‐sg*Fbn*1 in aorta, mice were preinfused with Ang II (or saline) for 7 days and then injected with CHO‐PGEA/pCas9‐sg*Fbn*1 via eye canthus intravenous for another 7 days (**Figure** **6**a). Of note, it was found that Ang II infusion significantly improved Cas9 expression at the aortic tissues (Figure 6b), suggesting the enrichment of nanoparticles in aorta. Inspired by above results, the aorta dilation and expression of Smad2/3‐target genes after 28‐day treatments with CHO‐PGEA/pCas9‐sg*Fbn*1 were evaluated under the Ang II infusion. The expression levels of *Mmp‐*2 and *Ctgf* were significantly increased (Figure 6c), indicating the successful *Fbn*1 gene disruption induced by Cas9‐sg*Fbn*1. Meanwhile, CHO‐PGEA/pCas9‐sg*Fbn*1 produced a significant increase in aortic diameter comparing with CHO‐PGEA/pCas9‐sgnull (Figure 6d,e).

### 2.6 In Vivo Safety Analysis

**Figure 7:**
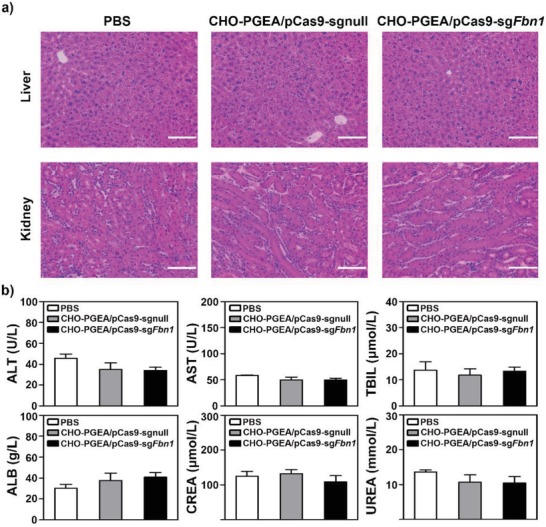
a) H&E staining of liver and kidney after Ang II infusion followed by CHO‐PGEA/pCas9‐sgnull and CHO‐PGEA/pCas9‐*Fbn*1 injection. The scale bar indicates 50 µm. b) Plasma chemistry profile analysis of alanine transaminase (ALT), aspartate transaminase (AST), total bilirubin (TBIL), albumin (ALB), Creatinine (CREA), and urea.

Safe delivery of CRISPR/Cas9 system is important for its in vivo applications. Therefore, the signs of toxicity or acute immune response in the CHO‐PGEA/pCas9‐sg*Fbn*1‐treated mice were examined. At the end of 28‐day treatment (Figure 6a), liver and kidney tissues were collected to conduct histological examination by hematoxylin and eosin (H&E) assay. There was no evidence of abnormal and inflammatory cell infiltration, such as aggregates of lymphocytes or macrophages (**Figure** **7**a). At the same time, blood chemistry profile analysis was also performed to assess the safety of in vivo nanoparticle delivery. No abnormal signals in all indicators of blood chemistry were detected (Figure 7b), which is consistent with the low cytotoxicity of CHO‐PGEA in vitro (Figure S6 in the Supporting Information). Together, these results indicated that CHO‐PGEA had no toxic effects on organs and had good potential for in vivo application.

## 3 Conclusions

In this work, it was demonstrated that hydroxyl‐rich gene carriers with cholesterol molecules (CHO‐PGEA) were suitable for CRISPR/Cas9 delivery to VSMCs and aorta. CHO‐PGEA could complex all‐in‐one CRISPR/Cas9 plasmid into stable nanoparticles, show good abilities of cellular uptake, endosomal escape, nuclear translocation, and thus produce efficient *Fbn*1 gene disruption in VSMCs. The in vivo studies indicated that Ang II infusion significantly increased the enrichment of CHO‐PGEA/Cas9 in aortic tissues. Furthermore, CHO‐PGEA/pCas9‐sg*Fbn*1 induced loss‐of‐function mutations of *Fbn*1 gene and caused aortic diameter dilation in vivo. The delivery system constructed here had no toxic effects on organs and showed good potential in vivo application. Given the potency of CHO‐PGEA/pCas9‐sg*Fbn*1 for aortic Cas9 delivery and gene editing in vivo, new candidate gene which was discovered from aortic aneurysm patients by whole exome sequencing could be edited. With the in vivo gene compilation experiments and pathological detection, this work could present an efficient strategy for identifying whether newly candidate genes discovered in aortic disease patients are pathogenic or benign.

## 4 Experimental Section

*Materials*: Branched PEI (*M*_w_ ≈25 kDa), angiotensin II (Ang II), and Cell Counting Kit‐8 (CCK8) were bought from Sigma‐Aldrich Chemical Co. (St Louis, MO, USA). Dulbecco's modified eagle medium (DMEM) was obtained from Hyclone Co. (St. Utah, Waltham, MA, USA) and fetal bovine serum (FBS) was purchased from Gibco Co. (Carlsbad, CA, USA). Lipofectamine 3000 reagent, YOYO‐1, Lyso Tracker red, DAPI, and TRIzol lysis buffer were purchased from Thermo Fisher Scientific (Carlsbad, CA, USA). SYBR Green PCR Master Mix was from Takara Bio (Takara, Otsu, Shiga, Japan). T7 Endonuclease I was from New England Biolabs. Anti‐Flag antibody was from Sigma‐Aldrich Chemical Co. (St Louis, MO, USA) and anti‐α‐SMA, anti‐GAPDH, anti‐p‐Smad2/3, and anti‐Smad2/3 antibodies were obtained from Abcam (Cambridge, MA, USA).

*Cell Culture*: Mouse neuroblastoma cell line Neuro‐2a (N2a) was purchased from Peking Union Medical College and maintained in DMEM supplemented with 10% FBS at 37 °C with 5% CO_2_. VSMCs were isolated from C57BL/6 mouse aorta and maintained in smooth muscle cell culture (ScienCell Research Laboratories, USA) supplemented with 10% FBS at 37 °C with 5% CO_2_.

*Design and Validation of Single Guide RNAs*: Candidate guide RNAs were designed using the optimized CRISPR design website: http://crispr.mit.edu/. Three candidate sgRNAs (sgRNA1, sgRNA2, sgRNA3) were selected. The sequences of sgRNAs are listed in Table S2 in the Supporting Information. Sense and antisense 24‐nt oligos for sgRNA cloning were annealed and ligated to the BbsI‐digested pX459 vector (Addgene). Each guide RNA plasmid was transfected into N2a cells using Lipofectamine 3000 reagent according to the manufacturer's instruction. Genomic DNA was extracted from the transfected cells at 72 h post transfection. PCR products of the targeted *Fbn*1 locus, which was amplified by the corresponding primer (*Fbn*1 F: 5′CAGTGTGCAGAGGCAAGGTATGAGAC3′, *Fbn*1 R: 5′CACCTGTGGCCACACTAATGACA3′), were purified and evaluated by T7EI cleavage assays according to the manufacturer's instructions. The final products were run on 2.0% agarose gels and analyzed with a Gel Doc gel imaging system (Bio‐Rad). The indel efficiency was calculated using the equation: (b+c)/(a+b+c) × 100, where a is uncut band, b and c are cut bands. The band intensity was quantified using Image J. PCR products were subjected to sanger sequencing and then subcloned into T‐clone vector pMD19‐T (Takara, Otsu, Shiga, Japan) for DNA sequencing. PCR products of the off‐targeted sites were amplified by the primer in Table S3 in the Supporting Information.

*Preparation and Characterization of CHO‐PGEA/pCas9‐sgFbn*1: CHO‐PGEA was synthesized based on CHO‐PGMA (Mn = 9.25 × 10^3^ g mol^−1^ with polydispersity index of 1.3) as we reported before.^30^ Polycation to pDNA ratios are expressed as molar ratios of nitrogen (N) in polymer to phosphate (P) in pDNA (or as N/P ratios). At different N/P ratios, equal volumes of polycation and pDNA solutions were mixed, and incubated for 30 min at room temperature to produce polycation/pDNA nanoparticles. The particle sizes and zeta‐potentials of the nanoparticles were investigated via dynamic light scattering (Malvern Nano‐ZS90). The morphology was imaged using an AFM with a Nanoscope IIIa controller (Veeco, Santa Barbara, CA, USA) at a setting of 512 pixels per line. Protein absorption and hemolysis assays of polycations were conducted according to our previous work.^44^

*Cas9 Plasmid Transfection In Vitro*: To evaluate the transfection efficiency of CHO‐PGEA/pCas9 nanoparticles, the plasmid pSpCas9(BB)‐2A‐GFP (pX458) carrying the 2A‐GFP fused to Cas9 expression cassette was used to allow the observation of Cas9 expression in transfected cells. VSMCs were seeded in 24‐well plates (5 × 10^4^ cells per well) and incubated for 24 h. Then CHO‐PGEA/pCas9‐GFP nanoparticles (1 µg per well of DNA in 20 µL H_2_O) at different N/P were added into the well. After 4 h incubation, the transfection medium was replaced with culture medium containing 10% FBS. After 24 h incubation, the cells were observed and measured using a confocal microscope (Zeiss LSM700) and flow cytometry analysis.

*In Vitro Cytotoxicity Assay*: The cytotoxicities of polycation/pDNA complexes were analyzed by CCK8 assays in VSMCs. Briefly, 1 × 10^4^ cells were seeded in 96‐well plates and incubated for 24 h. Then, the culture medium was replaced with the fresh medium which contained polycation/pDNA complexes under different N/P ratio, respectively. The culture medium in every well contained 0.25 µg of pDNA. After 4 h incubation, the transfection medium was replaced with fresh culture medium. After another 20 h incubation, 10 µL of CCK8 was added into each well. The final absorbance was recorded at a wavelength of 450 nm. The cell viability was expressed as the percentage relative to that of control.

*Cellular Uptake*: To analyze the cellular uptake of CHO‐PGEA/pCas9‐sg*Fbn*1, pCas9‐sg*Fbn*1 plasmids were labeled with YOYO‐1 according to the manufacturer's instruction followed by adding to CHO‐PGEA. VSMCs were seeded in a 24‐well plate (5 × 10^4^ cells per well) and cultured for 24 h before use. The cells were then treated with CHO‐PGEA/pCas9‐sg*Fbn*1‐YOYO‐1 nanoparticles at their optional N/P ratios. After 4 h incubation, the cells were collected for flow cytometry analysis.

*Endosomal Escape*: To detect the intracellular distribution of CHO‐PGEA/Cas9‐sg*Fbn*1, the plasmid was labeled with YOYO‐1. VSMCs were seeded on coverslips and incubated for 24 h and then treated with CHO‐PGEA nanoparticles loaded with 2 µg of Cas9‐sg*Fbn*1 plasmid. The Lyso Tracker Red was used to label lysosomes and endosomes according to the manufacturer's instruction. At determined time intervals, the cells were washed with PBS, fixed in 4% paraformaldehyde, counterstained with DAPI, and observed by a confocal microscope (Zeiss LSM700).

*In Vitro Cas9 Expression*: VSMCs were seeded in a 6‐well plate and cultured for 24 h. Then Cas9‐sg*Fbn*1 plasmid was transfected into the cells using CHO‐PGEA or PEI. For the detection of Cas9 mRNA levels, the transfected cells were collected at 24 h post‐transfection by using the TRIzol reagent to extract total RNAs. Then cDNA was synthesized using GoScriptTM Reverse Transcription System according to the manufacturer's protocol (Promega, Madison, WI, USA). The RT‐PCR was amplified with SYBR Green PCR Master Mix and run on a Bio‐Rad C1000 Touch Thermal Cycler (CFX384 Real‐time System). All experiments were measured in triplicate. Primers used here was shown in Table S4 in the Supporting Information.

For the detection of Cas9 expression at protein levels, the transfected cells were collected at 36 h post‐transfection and total proteins were extracted with cell lysis buffer for western blot analysis. Briefly, total proteins were separated by sodium dodecyl sulfate polyacrylamide gel eletrophoresis (SDS‐PAGE) and transferred to nitrocellulose membranes. After blocking with 5% milk, the membrane was incubated with the anti‐Flag (1:500) and anti‐GAPDH (1:500) antibodies at 4 °C overnight. After washing, the membranes were incubated with infrared dye‐conjugated secondary antibodies (1:10 000) for 1 h at room temperature. Antibody binding was detected using the Odyssey infrared imaging system (LI‐COR Biosciences, Lincoln, NE). The individual band intensity was quantified using Image J (NIH).

*In Vitro Fbn1 Gene Disruption*: VSMCs were transfected with Cas9‐sg*Fbn*1 plasmid with CHO‐PGEA reagent for 36 h. Total DNA was extracted for PCR amplification. The PCR products of pCas9‐sg*Fbn*1 targeted genomic locus were purified and evaluated by sanger sequencing assay individually to detect genomic mutations.

To further analyze whether the genome editing leads to *Fbn*1 loss‐of‐function, VSMCs were treated with CHO‐PGEA/pCas9‐sg*Fbn*1 for 72 h. Total RNAs were extracted for the analysis of expression levels of *Fbn*1 targeted genes *Mmp‐*2 and *Ctgf* by real‐time PCR assay with the primers listed in Table S4 (Supporting Information). Additionally, total proteins were prepared for evaluation of the effects of *Fbn*1 loss‐of‐function mutation on TGF‐β signaling pathway by using the western blot analysis for phosphorylation of Smad2/3.

*Nanoparticle Distribution and Cas9 Expression in Aorta*: 8‐week‐old male wild‐type C57BL/6J mice were received from Chinese Academy of Medical Sciences (Beijing, China). The mice were maintained in a pathogen‐free environment and at a controlled temperature. All animals' experiments were conducted in accordance with the Guide for Care and Use of Laboratory Animals, approved by the Animal Care and Use Committee of Capital Medical University. (Beijing, China). For in vivo imaging, Cas9‐sg*Fbn*1 plasmids were labeled by nucleic acid labeling kits‐Cy5 (Mirus Bio LLC) for living imaging (named pCas9‐Cy5). A total of 15 mice were divided into 5 groups as follows: 1) pCas9‐Cy5; 2) saline + PEI/pCas9‐Cy5; 3) Ang II + PEI/pCas9‐Cy5; 4) saline + CHO‐PGEA/pCas9‐Cy5; 5) Ang II + CHO‐PGEA/pCas9‐Cy5. Mice were first infused with saline or Ang II at 1500 ng kg^−1^ per min or infused with saline for 7 days and then treated with PEI/pCas9‐Cy5 (N/P ratio was 10) or CHO‐PGEA/pCas9‐Cy5 (N/P ratio was 15) nanoparticles via intravenous (i.v.) injection of eye canthus vein. Each injection dose of the complex solution containing 20 µg pCas9‐Cy5 was 100 µL. In control group, mice were treated with the solution containing 20 µg pCas9‐Cy5. After 1 h, all the mice were sacrificed and aortas were captured and analyzed by Xenogen IVIS spectrum (Caliper Life Science, America) with living image 2.11 software. Aorta sections were observed to detect the entry of polycation/pCas9‐Cy5 nanoparticles into mouse thoracic aortas. Briefly, the aorta section underwent immunofluorescence staining with the anti‐α‐SMA (1:100) at 4 °C overnight, then with secondary antibodies at 37 °C for 2 h. The images were captured by a confocal microscope (Zeiss LSM700).

For Cas9 expression in vivo, a total of 24 mice were divided into four groups as follows: 1) PBS; 2) CHO‐PGEA/pCas9‐sg*Fbn*1; 3) Ang II + PBS; 4) Ang II + CHO‐PGEA/pCas9‐sg*Fbn*1. Mice were first infused with Ang II at 1500 ng kg^−1^ per min or infused with saline for 7 days and then treated with CHO‐PGEA/pCas9‐sg*Fbn*1 at the N/P ratio of 15 via intravenous (i.v.) injection of eye canthus vein for another 7 days. RT‐PCR were used to analyse the levels of Cas9 with the primers listed in Table S4 in the Supporting Information.

*Fbn1 Disruption In Vivo*: A total of 24 mice were divided into two groups as follows: 1) CHO‐PGEA/pCas9‐sgnull (control); 2) CHO‐PGEA/pCas9‐sg*Fbn*1. The mice were first infused with Ang II followed by IV injection of CHO‐PGEA/pCas9‐sgnull or CHO‐PGEA/pCas9‐sg*Fbn*1 at the N/P ratio of 15 containing 40 µg of plasmid DNA. At 28 days after the nanoparticle injection, six mice randomly selected from each group were euthanized, and aortic arteries were collected. The total RNAs were extracted from aortic artery, and RT‐PCR analysis was preformed to detect the expression of *Fbn*1 targeted genes *Mmp‐*2 and *Ctgf*. Meanwhile another six mice were euthanized, and aortic arteries were collected. H&E staining was used to measure the diameter of thoracic aorta according to the manufacturer's instruction.

For safety evaluation, the mice were infused with Ang II followed by i.v. injection of nanoparticles. After 28 days, the liver and kidney tissues were collected, embedded in paraffin, sectioned (5 µm), and stained with H&E to detect the liver and kidney toxicity induced by CHO‐PGEA/plasmid nanoparticles. Plasma was collected for biochemical measurement of alanine transaminase (ALT), aspartate transaminase (AST), albumin (ALB), total bilirubin (TBIL), UREA, and creatinine (CREA) using an autoanalyzer (RA 1000; Technicon Instruments, NY).

*Statistical Analysis*: All data are presented as means ± standard deviations and are representative of at least three individual experiments. Statistical analyses were conducted using SPSS 24 software with a Student's *t*‐test for two‐sample analyses and one‐way ANOVA for multiple sample analyses. In all tests, significant differences were indicated by *P < 0.05, **P < 0.01, and ***P < 0.001.

## Conflict of Interest

The authors declare no conflict of interest.

## Supporting information

Supplementary

## References

[1] K. Suzuki , Y. Tsunekawa , R. Hernandez‐Benitez , J. Wu , J. Zhu , E. J. Kim , F. Hatanaka , M. Yamamoto , T. Araoka , Z. Li , M. Kurita , T. Hishida , M. Li , E. Aizawa , S. Guo , S. Chen , A. Goebl , R. D. Soligalla , J. Qu , T. Jiang , X. Fu , M. Jafari , C. R. Esteban , W. T. Berggren , J. Lajara , E. Nunez‐Delicado , P. Guillen , J. M. Campistol , F. Matsuzaki , G. H. Liu , P. Magistretti , K. Zhang , E. M. Callaway , K. Zhang , J. C. Belmonte , Nature 2016, 540, 144. 27851729 10.1038/nature20565 PMC5331785

[2] W. Xue , S. Chen , H. Yin , T. Tammela , T. Papagiannakopoulos , N. S. Joshi , W. Cai , G. Yang , R. Bronson , D. G. Crowley , F. Zhang , D. G. Anderson , P. A. Sharp , T. Jacks , Nature 2014, 514, 380. 25119044 10.1038/nature13589 PMC4199937

[3] C. Long , L. Amoasii , A. A. Mireault , J. R. McAnally , H. Li , E. Sanchez‐Ortiz , S. Bhattacharyya , J. M. Shelton , R. Bassel‐Duby , E. N. Olson , Science 2016, 351, 400. 26721683 10.1126/science.aad5725 PMC4760628

[4] P. Mali , L. Yang , K. M. Esvelt , J. Aach , M. Guell , J. E. DiCarlo , J. E. Norville , G. M. Church , Science 2013, 339, 823. 23287722 10.1126/science.1232033 PMC3712628

[5] P. D. Hsu , E. S. Lander , F. Zhang , Cell 2014, 157, 1262. 24906146 10.1016/j.cell.2014.05.010 PMC4343198

[6] A. Singh , D. Chakraborty , S. Maiti , Chem. Soc. Rev. 2016, 45, 6666. 27711765 10.1039/c6cs00197a

[7] B. Steyer , J. Carlson‐Stevermer , N. Angenent‐Mari , A. Khalil , T. Harkness , K. Saha , Acta Biomater. 2016, 34, 143. 26747759 10.1016/j.actbio.2015.12.036 PMC4961091

[8] Y. I. Jo , B. Suresh , H. Kim , S. Ramakrishna , Biochim. Biophys. Acta 2015, 1856, 234. 26434948 10.1016/j.bbcan.2015.09.003

[9] K. Lee , M. Conboy , H. M. Park , F. Jiang , H. J. Kim , M. A. Dewitt , V. A. Mackley , K. Chang , A. Rao , C. Skinner , T. Shobha , M. Mehdipour , H. Liu , W. C. Huang , F. Lan , N. L. Bray , S. Li , J. E. Corn , K. Kataoka , J. A. Doudna , I. Conboy , N. Murthy , Nat. Biomed. Eng. 2017, 1, 889. 29805845 10.1038/s41551-017-0137-2 PMC5968829

[10] C. S. Young , M. R. Hicks , N. V. Ermolova , H. Nakano , M. Jan , S. Younesi , S. Karumbayaram , C. Kumagai‐Cresse , D. Wang , J. A. Zack , D. B. Kohn , A. Nakano , S. F. Nelson , M. C. Miceli , M. J. Spencer , A. D. Pyle , Cell Stem Cell 2016, 18, 533. 26877224 10.1016/j.stem.2016.01.021 PMC4826286

[11] J. Liao , R. Karnik , H. Gu , M. J. Ziller , K. Clement , A. M. Tsankov , V. Akopian , C. A. Gifford , J. Donaghey , C. Galonska , R. Pop , D. Reyon , S. Q. Tsai , W. Mallard , J. K. Joung , J. L. Rinn , A. Gnirke , A. Meissner , Nat. Genet. 2015, 47, 469. 25822089 10.1038/ng.3258 PMC4414868

[12] W. Yu , S. Mookherjee , V. Chaitankar , S. Hiriyanna , J. W. Kim , M. Brooks , Y. Ataeijannati , X. Sun , L. Dong , T. Li , A. Swaroop , Z. Wu , Nat. Commun. 2017, 8, 14716. 28291770 10.1038/ncomms14716 PMC5355895

[13] L. Li , L. Song , X. Liu , X. Yang , X. Li , T. He , N. Wang , S. Yang , C. Yu , T. Yin , Y. Wen , Z. He , X. Wei , W. Su , Q. Wu , S. Yao , C. Gong , Y. Wei , ACS Nano 2017, 11, 95. 28114767 10.1021/acsnano.6b04261

[14] H. Yin , C. Q. Song , J. R. Dorkin , L. J. Zhu , Y. Li , Q. Wu , A. Park , J. Yang , S. Suresh , A. Bizhanova , A. Gupta , M. F. Bolukbasi , S. Walsh , R. L. Bogorad , G. Gao , Z. Weng , Y. Dong , V. Koteliansky , S. A. Wolfe , R. Langer , W. Xue , D. G. Anderson , Nat. Biotechnol. 2016, 34, 328. 26829318 10.1038/nbt.3471 PMC5423356

[15] J. A. Zuris , D. B. Thompson , Y. Shu , J. P. Guilinger , J. L. Bessen , J. H. Hu , M. L. Maeder , J. K. Joung , Z. Y. Chen , D. R. Liu , Nat. Biotechnol. 2015, 33, 73. 25357182 10.1038/nbt.3081 PMC4289409

[16] Y. Yang , L. Wang , P. Bell , D. McMenamin , Z. He , J. White , H. Yu , C. Xu , H. Morizono , K. Musunuru , M. L. Batshaw , J. M. Wilson , Nat. Biotechnol. 2016, 34, 334. 26829317 10.1038/nbt.3469 PMC4786489

[17] P. Wang , L. Zhang , W. Zheng , L. Cong , Z. Guo , Y. Xie , L. Wang , R. Tang , Q. Feng , Y. Hamada , K. Gonda , Z. Hu , X. Wu , X. Jiang , Angew. Chem., Int. Ed. 2018, 57, 1491. 10.1002/anie.201708689 29282854

[18] Z. Chen , F. Liu , Y. Chen , J. Liu , X. Wang , A. T. Chen , G. Deng , H. Zhang , J. Liu , Z. Hong , J. Zhou , Adv. Funct. Mater. 2017, 27, 1703036. 29755309 10.1002/adfm.201703036 PMC5939593

[19] Y. Lin , J. Wu , W. Gu , Y. Huang , Z. Tong , L. Huang , J. Tan , Adv. Sci. 2018, 5, 1700611. 10.1002/advs.201700611 PMC5908366 29721412

[20] H. X. Wang , Z. Song , Y. H. Lao , X. Xu , J. Gong , D. Cheng , S. Chakraborty , J. S. Park , M. Li , D. Huang , L. Yin , J. Cheng , K. W. Leong , Proc. Natl. Acad. Sci. USA 2018, 115, 4903. 29686087 10.1073/pnas.1712963115 PMC5948953

[21] S. Lee , H. Koo , J. H. Na , S. J. Han , H. S. Min , S. J. Lee , S. H. Kim , S. H. Yun , S. Y. Jeong , I. C. Kwon , K. Choi , K. Kim , ACS Nano 2014, 8, 2048. 24499346 10.1021/nn406584y

[22] J. G. Pickering , J. Jekanowski , L. Weir , S. Takeshita , D. W. Losordo , J. M. Isner , Circulation 1994, 89, 13. 8281638 10.1161/01.cir.89.1.13

[23] S. Armeanu , J. Pelisek , E. Krausz , A. Fuchs , D. Groth , R. Curth , O. Keil , J. Quilici , P. H. Rolland , R. Reszka , S. Nikol , Mol. Ther. 2000, 1, 366. 10933955 10.1006/mthe.2000.0053

[24] L. Song , N. Zhao , F. J. Xu , Adv. Funct. Mater. 2017, 27, 1701255.

[25] Y. Sun , H. Hu , N. Zhao , T. Xia , B. Yu , C. Shen , F. J. Xu , Biomaterials 2017, 117, 77. 27939903 10.1016/j.biomaterials.2016.11.055

[26] F. J. Xu , Prog. Polym. Sci. 2018, 78, 56.

[27] R. Q. Li , Y. Wu , Y. Zhi , X. Yang , Y. Li , F. J. Xu , J. Du , Adv. Mater. 2016, 28, 7204. 27297033 10.1002/adma.201602319

[28] Y. Qi , H. Song , H. Xiao , G. Cheng , B. Yu , F. J. Xu , Small 2018, 14, 1803061. 10.1002/smll.201803061 30238691

[29] H. Song , W. Pan , R. Q. Li , B. Yu , W. Liu , M. Yang , F. J. Xu , Small 2018, 14, 1703152. 10.1002/smll.201703152 29280338

[30] C. Xu , B. Yu , H. Hu , M. N. Nizam , W. Yuan , J. Ma , F. J. Xu , Biomater. Sci. 2016, 4, 1233. 27374783 10.1039/c6bm00360e

[31] J. Sun , L. Zhang , J. Wang , Q. Feng , D. Liu , Q. Yin , D. Xu , Y. Wei , B. Ding , X. Shi , X. Jiang , Adv. Mater. 2015, 27, 1402. 25529120 10.1002/adma.201404788

[32] P. Wang , L. Zhang , Y. Xie , N. Wang , R. Tang , W. Zheng , X. Jiang , Adv. Sci. 2017, 4, 1700175. 10.1002/advs.201700175 PMC5700650 29201613

[33] A. Verstraeten , M. Alaerts , L. L. Van , B. Loeys , Hum. Mutat. 2016, 37, 524. 26919284 10.1002/humu.22977

[34] G. Pepe , B. Giusti , E. Sticchi , R. Abbate , G. F. Gensini , S. Nistri , Appl. Clin. Genet. 2016, 9, 55. 27274304 10.2147/TACG.S96233 PMC4869846

[35] S. A. LeMaire , M. L. McDonald , D. C. Guo , L. Russell , C. C. Miller , R. J. Johnson , M. R. Bekheirnia , L. M. Franco , M. Nguyen , R. E. Pyeritz , J. E. Bavaria , R. Devereux , C. Maslen , K. W. Holmes , K. Eagle , S. C. Body , C. Seidman , J. G. Seidman , E. M. Isselbacher , M. Bray , J. S. Coselli , A. L. Estrera , H. J. Safi , J. W. Belmont , S. M. Leal , D. M. Milewicz , Nat. Genet. 2011, 43, 996. 21909107 10.1038/ng.934 PMC3244938

[36] A. Granata , F. Serrano , W. G. Bernard , M. McNamara , L. Low , P. Sastry , S. Sinha , Nat. Genet. 2017, 49, 97. 27893734 10.1038/ng.3723

[37] C. Park‐Windhol , P. A. D'Amore , Annu. Rev. Pathol.: Mech. Dis. 2016, 11, 251. 10.1146/annurev-pathol-012615-044506 PMC8462517 26907525

[38] B. Williams , A. Q. Baker , B. Gallacher , D. Lodwick , Hypertension 1995, 25, 913. 7737726 10.1161/01.hyp.25.5.913

[39] A. Benigni , P. Cassis , G. Remuzzi , EMBO Mol. Med. 2010, 2, 247. 20597104 10.1002/emmm.201000080 PMC3377325

[40] M. Hadjidemetriou , K. Kostarelos , Nat. Nanotechnol. 2017, 12, 288. 28383044 10.1038/nnano.2017.61

[41] M. Tonigold , J. Simon , D. Estupinan , M. Kokkinopoulou , J. Reinholz , U. Kintzel , A. Kaltbeitzel , P. Renz , M. P. Domogalla , K. Steinbrink , I. Lieberwirth , D. Crespy , K. Landfester , V. Mailander , Nat. Nanotechnol. 2018, 13, 862. 29915272 10.1038/s41565-018-0171-6

[42] B. Wang , C. He , C. Tang , C. Yin , Biomaterials 2011, 32, 4630. 21440295 10.1016/j.biomaterials.2011.03.003

[43] A. O. Elzoghby , W. M. Samy , N. A. Elgindy , J. Controlled Release 2012, 161, 38. 10.1016/j.jconrel.2012.04.036 22564368

[44] Y. Huang , X. Ding , Y. Qi , B. Yu , F. J. Xu , Biomaterials 2016, 106, 134. 27561884 10.1016/j.biomaterials.2016.08.025

[45] Z. Isogai , R. N. Ono , S. Ushiro , D. R. Keene , Y. Chen , R. Mazzieri , N. L. Charbonneau , D. P. Reinhardt , D. B. Rifkin , L. Y. Sakai , J. Biol. Chem. 2003, 278, 2750. 12429738 10.1074/jbc.M209256200

[46] P. T. Dijke , H. M. Arthur , Nat. Rev. Mol. Cell Biol. 2007, 8, 857. 17895899 10.1038/nrm2262

[47] E. R. Neptune , P. A. Frischmeyer , D. E. Arking , L. Myers , T. E. Bunton , B. Gayraud , F. Ramirez , L. Y. Sakai , H. C. Dietz , Nat. Genet. 2003, 33, 407. 12598898 10.1038/ng1116

[48] T. M. Holm , J. P. Habashi , J. J. Doyle , D. Bedja , Y. Chen , C. V. Erp , M. E. Lindsay , D. Kim , F. Schoenhoff , R. D. Cohn , B. L. Loeys , C. J. Thomas , S. Patnaik , J. J. Marugan , D. P. Judge , H. C. Dietz , Science 2011, 332, 358. 21493862 10.1126/science.1192149 PMC3111087

