# Supplementary material for: CRISPR/Cas9 Delivery Mediated with Hydroxyl‐Rich Nanosystems for Gene Editing in Aorta
Source: Adv Sci (Weinh). 2019 Apr 20;6(12):1900386. doi: 10.1002/advs.201900386 (PMC6662060; doi:10.1002/advs.201900386)
Supplement: Supplementary file 1 — Supplementary [file ADVS-6-1900386-s001.pdf]

Copyright WILEY-VCH Verlag GmbH & Co. KGaA, 69469 Weinheim, Germany, 2019.

ADVANCED SCIENCE
Open Access

Supporting Information

for *Adv. Sci.,* DOI: 10.1002/advs.201900386

CRISPR/Cas9 Delivery Mediated with Hydroxyl-Rich Nanosystems for Gene Editing in Aorta

Xiaoping Zhang, Chen Xu, Shijuan Gao, Ping Li, Yu Kong, Tiantian Li, Yulin Li, Fu-Jian Xu,* and Jie Du**

Supporting Information

CRISPR/Cas9 Delivery Mediated with Hydroxyl-Rich Nanosystems for Gene Editing in Aorta

Xiaoping Zhang,[†] *Chen Xu,*[†] *Shijuan Gao, Ping Li, Yu Kong, Tiantian Li, Yulin Li*[*]*,*

Fu-Jian Xu, and Jie Du**

X. P. Zhang, C. Xu, T. T. Li, Prof. F-J. Xu
State Key Laboratory of Chemical Resource Engineering, Key Lab of Biomedical Materials of Natural Macromolecules (Beijing University of Chemical Technology), Ministry of Education, Beijing Laboratory of Biomedical Materials, and Beijing Advanced Innovation Center for Soft Matter Science and Engineering, Beijing University of Chemical Technology, Beijing 100029, China
E-mail: xufj@mail.buct.edu.cn (F-J. Xu)
Prof S. J. Gao, P. Li, Y. Kong, Prof. Y. L. Li, Prof. J. Du
Key Laboratory of Remodeling-Related Cardiovascular Diseases (Ministry of Education), and Beijing Institute of Heart, Lung, and Blood Vessel Diseases, Beijing Anzhen Hospital Affiliated to Capital Medical University, Beijing 100029, China
E-mail: jiedu@ccmu.edu.cn (J. Du); lyllyl_1111@163.com (Y. L. Li)
†Both authors contributed equally to this work.

Table S1 | PDI values of complexes

**Samples**	**Average PDI[a] (at different N/P ratios)**			
	**5**	**10**	**15**	**20**
PEI/pCas9-sg*Fbn1*		0.233		
CHO-PGEA/pCas9-sg*Fbn1*	0.229	0.158	0.150	0.137

[a]Determined from DLS results. PDI =Polydispersity Index.

Table S2. Sequences of sgRNA oligos

sgRNA	Oligo F	Oligo R
sgRNA1	5’CACCGGTTGCGATCTTGGCCGGTGC3’	5’AAACGCACCGGCCAAGATCGCAACC3’
sgRNA2	5’CACCGATGCAGTGCTGTTGCGATCT3’	5’AAACAGATCGCAACAGCACTGCATC3’
sgRNA3	5’CACCGAGTGCTGTTGCGATCTTGGC3’	5’AAACGCCAAGATCGCAACAGCACTC3’

Table S3. Oligo sequences for off-target detection

Gene name	Sequence 5'-3'	Primer F 5'-3'	Primer R 5'-3'
Neblin	GTTGCTCTTGTGATCTTGGCCAG	GCCACCTCATTCTGTCACCTACCAC	GGTGCAGTACGTGAGAGACTTACGTCAC
Nav	GCTGCTGTTGCGATCCTGGGTGG	TCAGTCACCTTAGGAGTACACCGCAC	GTCGGAAATGTGCCTTTCAGACAGC
Ndu	TGTGCTGTTCCGATCCGGGCCGG	GAAGGAATATGCCGTCCGCTAC	CGTGGGAGACAAATGAGGCGAA
Ttl	AGTGCTGTGGAGACCTTGGGAAG	TTCGTCAAGCTGTGAAGCACCGGA	CATCCAAGGCCAGAAACCGCTCAAAG

Table S4. The primer sequences for real-time PCR

Gene name	Primer F	Primer R
GAPDH	5’TGATGACATCAAGAAGGTGGTGAAG3’	5’TCCTTGGAGGCCATGTAGGCCAT3’
Mmp-2	5’TGGAATGCCATCCCTGATAA3’	5’AGCCCAGCCAGTCTGATTTG3’
Ctgf	5’TTGAAGTCTCAGAAGGTGGAT3’	5’GCAGGAGGTCGTAGGTCAC3’
Cas9	5’ATGGACTATAAGGACCACGACGGAGAC3’	5’CGGTGTTGCCCAGCACCTTGAAT3’

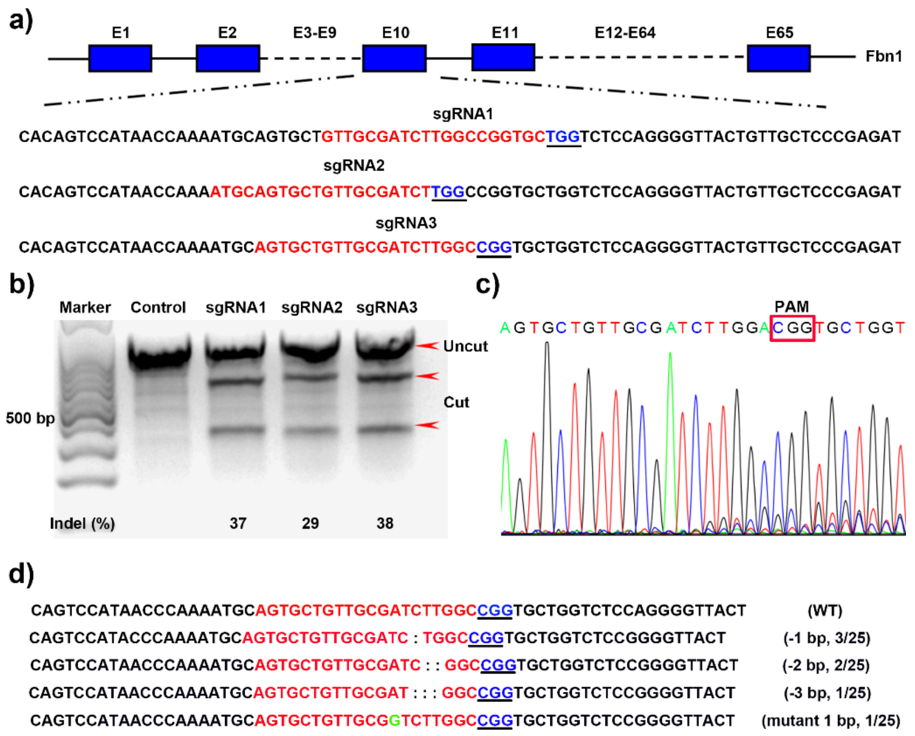

Figure S1. a) Schematic diagram of the candidate sgRNAs targeting *Fbn1* locus. The sgRNA pairs with the 20-bp DNA target (Red), upstream of 5’-NGG adjacent motif PAM (blue) and guides Cas9 nuclease to mediate a break 3 bp upstream of the PAM. b) Detection of Cas9/sgRNA-mediated cleavage of *Fbn1* loci by T7 endonuclease Ⅰ -based assay. c) Sanger sequencing of the PCR amplicon of the targeted sites in the N2a cells transfected with pCas9-sg*Fbn1* by Lipofectamine 3000. d) Representative sequences of *Fbn1* locus in pCas9-sg*Fbn1* transfected N2a cells.

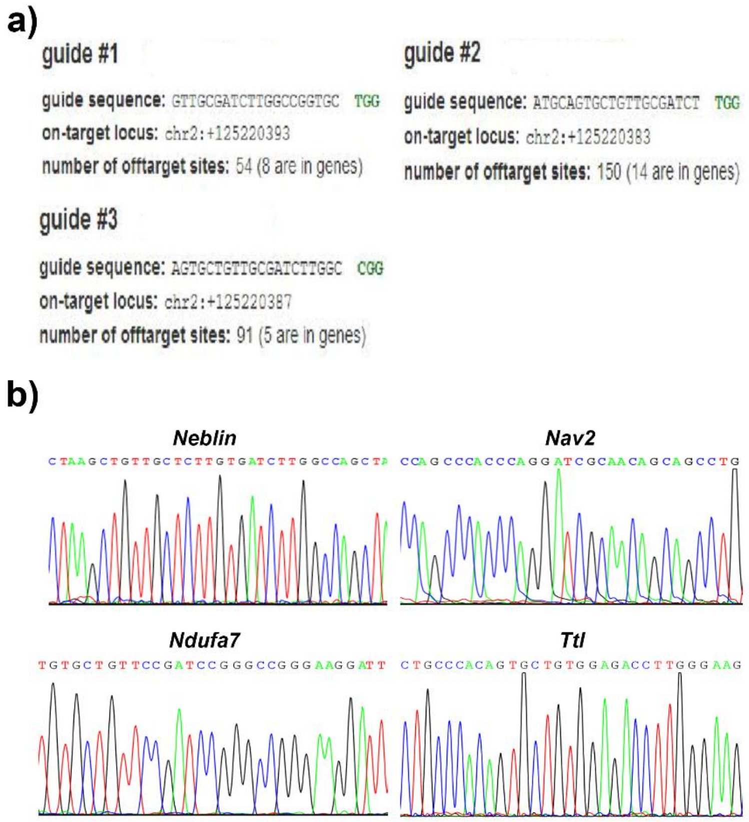

Figure S2. a) The screenshot of the number of off-target sites for the three sgRNAs predicted by the CRISPR design website: http://crispr.mit.edu/. b) Sanger sequencing of the potential off-targeted sites of sg*Fbn1* that are in open reading frames of *Neblin*, *Nav2*, *Ndufa7* and *Ttl* genes after pCas9-sg*Fbn1* transfection.

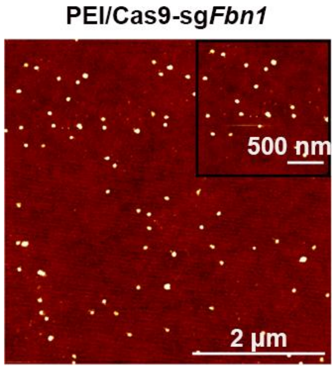

Figure S3. Morphology of PEI/pCas9-sg*Fbn1* nanoparticles at N/P=10 measured by atomic force microscope (AFM).

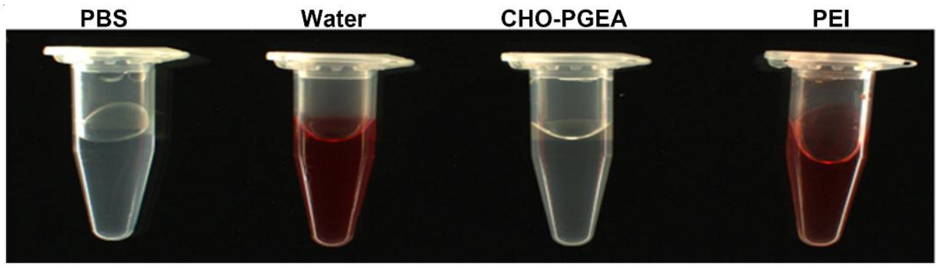

Figure S4. Images of RBCs treated with PBS, water, PEI/pCas9-sg*Fbn1* and CHO-PGEA/pCas9-sg*Fbn1* nanoparticles at their respective N/P ratios at the concentration of 1 mg/mL, where deionized water and PBS were used as the positive and negative controls.

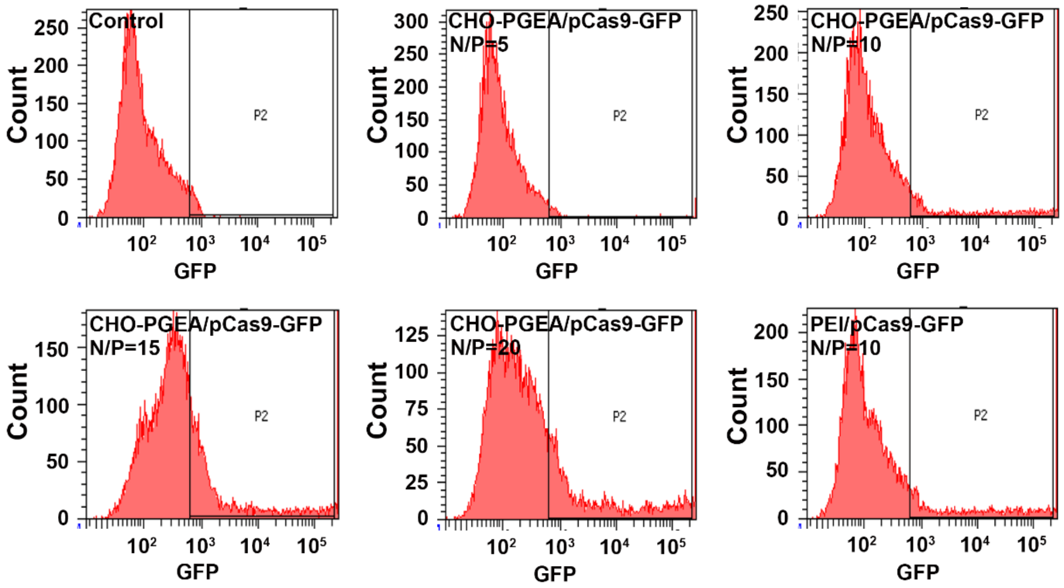

Figure S5. Transfection efficiencies of CHO-PGEA/pCas9-GFP at different N/P ratios and PEI/pCas9-GFP at the N/P ratio of 10 in mouse vascular smooth muscle cells (VSMCs).

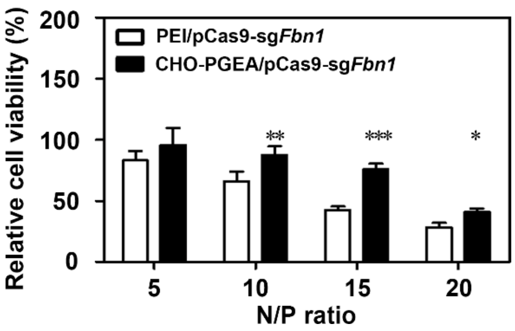

Figure S6. Cell viabilities of CHO-PGEA/pCas9-sg*Fbn1* and PEI/pCas9-sg*Fbn1* nanoparticles at various N/P ratios in VSMCs. Error bars represent the standard deviation of six measurements (*P < 0.05, **P < 0.01, ***P < 0.001).

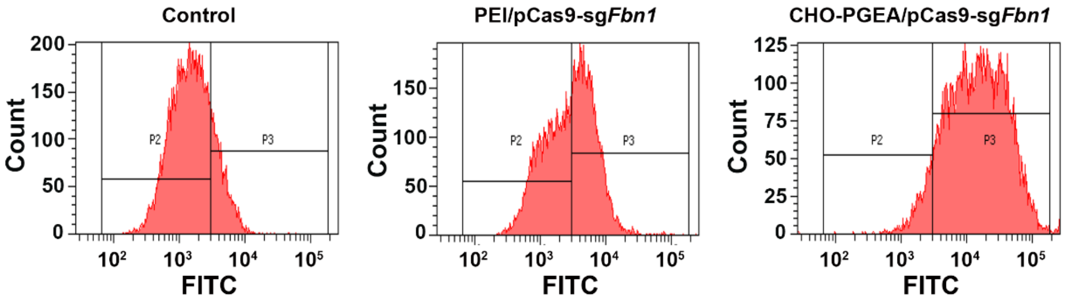

Figure S7. Flow cytometry analysis for cellular uptake of CHO-PGEA and PEI at their optimal N/P ratios.

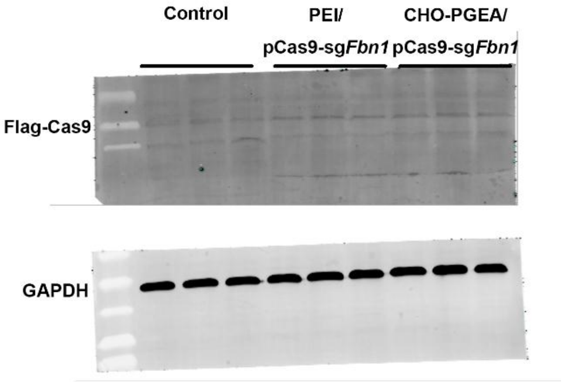

Figure S8. Raw data of western blot analysis of Cas9 protein expression in VSMCs incubated with nanoparticles with an anti-Flag antibody.

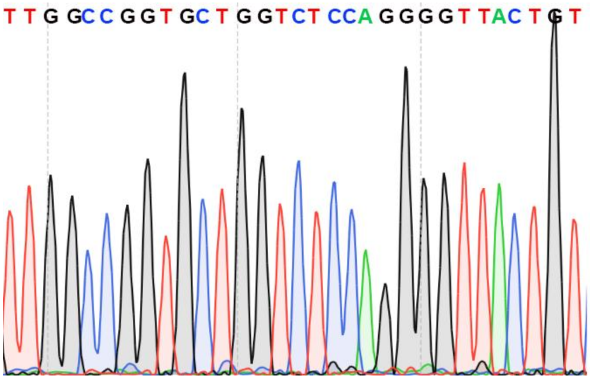

Figure S9. Sanger sequencing of the PCR amplicon of the targeted *Fbn1* locus in CHO-PGEA/pCas9-sgnull treated VSMCs.

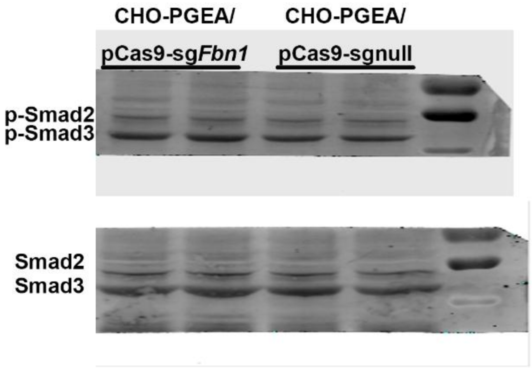

Figure S10. Raw data of western blot analysis of p-Smad2/3 and Smad2/3 expression in the VSMCs after CHO-PGEA/pCas9-sgnull and CHO-PGEA/pCas9-sg*Fbn1* treatment.

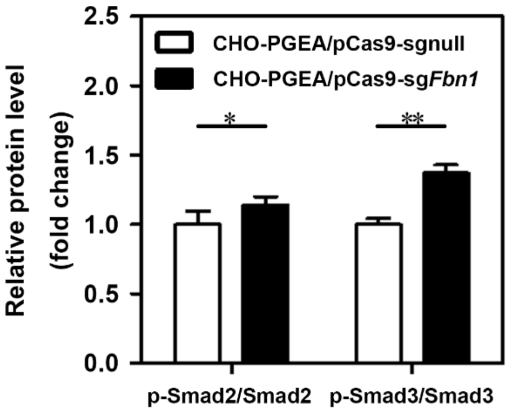

Figure S11. Quantification of relative protein levels (calculated from the relative density of the western blot bands) of p-Smad2/3 and Smad2/3 in the VSMCs after CHO-PGEA/pCas9-sgnull and CHO-PGEA/pCas9-sg*Fbn1* treatment. (**P* < 0.05, ***P* < 0.01).
